# Exosomal MiRNAs in Osteosarcoma: Biogenesis and Biological Functions

**DOI:** 10.3389/fphar.2022.902049

**Published:** 2022-05-03

**Authors:** Jinxin Tang, Jieyu He, Chengyao Feng, Chao Tu

**Affiliations:** ^1^ Department of Orthopaedics, The Second Xiangya Hospital, Central South University, Changsha, China; ^2^ Xiangya School of Medicine, Central South University, Changsha, China; ^3^ Department of Geriatrics, The Second Xiangya Hospital, Central South University, Changsha, China; ^4^ Hunan Key Laboratory of Tumor Models and Individualized Medicine, The Second Xiangya Hospital, Central South University, Changsha, China

**Keywords:** osteosarcoma, exosome, miRNA, non-coding RNA, biogenesis

## Abstract

MiRNAs are a group of non-coding RNA molecules that function in mRNA translational inhibition *via* base-pairing with complementary sequences in target mRNA. In oncology, miRNAs have raised great attention due to their aberrant expression and pivotal roles in the pathogenesis of multiple malignancies including osteosarcoma. MiRNAs can be transported by exosome, the nano-extracellular vesicle with a diameter of 30–150 nm. Recently, a growing number of studies have demonstrated that exosomal miRNAs play a critical role in tumor initiation and progression, by exerting multiple biological functions including metastasis, angiogenesis, drug resistance and immunosuppression. In this review, we aim to depict the biogenesis of exosomal miRNAs and summarize the potential diagnostic and therapeutic functions of exosomal miRNAs in osteosarcoma.

## 1 Introduction

Osteosarcoma is one of the primary bone malignancies that always occur in children, adolescents and young adults (Kallen and Hornick, 2021). It is an aggressive tumor that comes from primitive transformed cells of mesenchymal origin that exhibits osteoblastic differentiation and produces malignant osteoid (Luetke et al., 2014). Patients with osteosarcoma often die because of the pulmonary metastasis (Yang et al., 2020). Till now, due to the lack of efficient early diagnosis, the 5-year-survival rate and prognosis of osteosarcoma patients are still unsatisfactory. Tissue biopsy keeps representing the first choice for tumor diagnosis. But for pediatric patients, collecting accessible tissue can be hard. Even when tissues can be collected, the invasive procedure may increase the pressure on patient care, and impede repeated sampling (Ilié and Hofman, 2016). Accordingly, liquid biopsy by using circulating DNA, RNA, non-coding RNAs (ncRNAs), or soluble proteins, has become more attractive in osteosarcoma diagnosis.

Exosomes are nano-extracellular vesicles (EVs) with diameters ranging from 30 to 150 nm. They are widely and stably distributed in the majority of biological fluids including serum, plasma, urine, saliva, synovial fluid and cerebrospinal fluid (Chen et al., 2021; Thakur et al., 2021). It is now known that various metabolically active cells, such as neoplastic cells, stromal cells, macrophages, fibroblasts or other cells in tumor microenvironment, could release exosomes (Galardi et al., 2019). Over the past decade, it has been increasingly confirmed that exosomes play a central role in carcinogenesis by transporting bioactive molecules among these cells. The cargoes loaded in exosomes include proteins, lipids, peptide compounds, DNA, and microRNA (miRNA) (Colletti et al., 2017; Zheng et al., 2020). Among them, miRNAs have raised global attention due to their aberrant expressions and multifaceted biological functions in carcinogenesis, including angiogenesis, cell cycle, stemness, apoptosis, etc.

MiRNAs are a group of ncRNA molecules that function as either oncogene or tumor suppressor via base-pairing with complementary sequences in target mRNA at post-transcriptional level (Bartel, 2009; Rupaimoole and Slack, 2017). MiRNAs could exert their functions by interacting with other ncRNAs, including long non-coding RNAs (lncRNAs), pseudogene transcripts, and circular RNA (circRNA) (Tu et al., 2020a). Recently, the expression profile of various exosomal miRNAs has been found to be significantly associated with tumor aggressiveness. Accumulative studies have suggested a great potential of exosomal miRNAs in liquid biopsy for diagnosis and treatment prognosis of cancers, including osteosarcoma (Iorio and Croce, 2012). The purpose of this review is to briefly summarize the biogenesis of exosomal miRNAs and their physiological, pathological functions as well as potential clinical applications in osteosarcoma.

## 2 Exosomal MicroRNA: Biogenesis

### 2.1 Exosome

Exosomes originate from two main phases. Firstly, the intramembranous budding of plasma membrane forms early endosomes, then the endosomal membrane invaginates into surrounding lumina with biomolecules to form intraluminal vesicle (ILVs). Multivesicular bodies (MVBs) are later endosomes with dozens of ILVs. Some of these MVBs are fused with autophagosomes to generate amphisomes, and finally combine with lysosomes to degrade the cargos wrapped inside, which can inhibit the transport of cargos like miRNAs and proteins through exosomes (Fader and Colombo, 2009). While the other MVBs are eventually transported and fused with the plasma membrane to release exosomes (Yue et al., 2020). The endosomal sorting complexes required for transport (ESCRT) machinery is critical in this process. ESCRT comprises several complexes, ESCRT-0, -I, -II, -III, and some associated proteins like TSG101, ALIX and VPS4. ESCRT-0 (Hrs, a component of ESCRT-0) contributes to the sorting of cargoes into exosomes. ESCRT-I and II help to induce the deformation of membrane to form stable membrane neck. ESCRT-III plays an important part in vesicle neck scission and membrane remodeling (Hessvik and Llorente, 2018; McAndrews and Kalluri, 2019). In this ESCRT-dependent process, ESCRT-0 firstly recognizes mono-ubiquitinated proteins by virtue of Hrs heterodimer (McGough and Vincent, 2016). Afterwards, ESCRT-I and II are raised to generate a sorting domain with great affinity to ubiq-uitylated cargos and prepare for the membrane deformation (Mashouri et al., 2019). Finally, ESCRT- III binds to the complex and drives membrane deformation to release the buds into the endosome (Vietri et al., 2020).

Except the ESCRT-dependent pathway, experiments have also shown the existence of ESCRT-independent pathway as well. Wei DH et al. found that RAB31, a member of RAS oncogene family, controls an ESCRT-independent exosome pathway. RAB31 activated by epidermal growth factor receptor (EGFR) can direct EGFR localization to CD63^+^ multivesicular endosomes (MVEs) and induce the formation of ILVs and exosomes. Meanwhile, RAB31 can also recruit TBC1 domain family member 2B (TBC1D2B) to de-activate RAB7 to inhibit the fusion of MVEs with lysosomes and assist the exosomes secretion (Wei et al., 2021).

### 2.2 MicroRNA

MiRNAs are small endogenous single-stranded RNAs that contain about 19–25 nucleotides in length without protein-coding capacity. MiRNA genes are transcribed by two RNA polymerases, namely, the pol II and pol III. The results of the transcription are some precursors of miRNAs called primary miRNAs (pri-miRNAs) that contain a hairpin structure where miRNA sequences are inserted (Ha and Kim, 2014). Following transcription, the pri-miRNAs go through several steps of maturation to form precursor miRNAs (pre-miRNAs) in the nuclear with the function of nuclear RNase III Drosha and its cofactor DiGeorge syndrome critical region 8 (DGCR8) (Lee et al., 2003). Then the processed pre-miRNAs are transported by a complex formed with exportin-5 and GTP-binding nuclear protein RAN•GTP from the nuclear to the cytosol, in which the pre-miRNAs will be cut into short segments by the protein Dicer and finally generate a mature miRNA (Bartel, 2004).

### 2.3 Exosomal MicroRNAs

**FIGURE 1:**
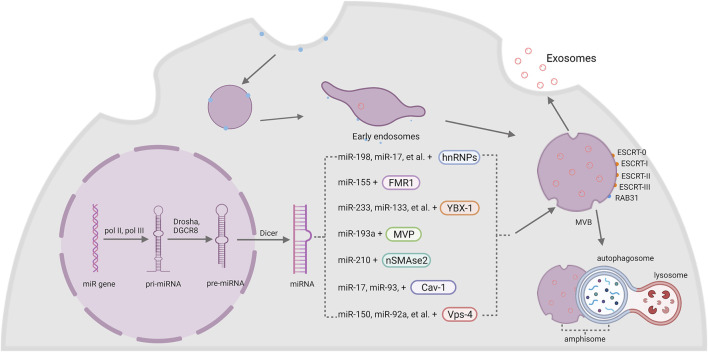
The biogenesis of exosomal miRNA. The intramembranous budding of plasma membrane forms exosomes with the help of RAB31 or endosomal sorting complexes required for transport (ESCRT-0, -I, -II, -III). MiRNA genes are transcribed into pri-miRNA by pol II and pol III, then Drosha and DGCR8 modify it into pre-miRNA and generate a mature miRNA with the help of Dicer. Finally, miRNAs are sorted into exosomes by two kinds of membrane: RBPs and membrane proteins, including hnRNPs, FMR1, YBX-1, MVP, nSMase2, Cav-1 and Vps-4.

Cells have special mechanisms to selectively sort miRNAs into exosomes, and subsequently transmit specific information between each other. Basically, proteins that participating in the sorting of miRNAs can be classified into two categories: RNA-binding proteins (RBPs) and membrane proteins (Groot and Lee, 2020), (Figure 1).

#### 2.3.1 RNA-Binding Proteins

Heterogeneous nuclear ribonucleoproteins (hnRNPs) are a group of RBPs that assist with the sorting process of miRNAs into exosomes. The hnRNPA2B1 protein was found to bind miR-198, miR-17, miR-93 via recognizing AGG/UAG motifs (Villarroya-Beltri et al., 2013; Wu et al., 2018). Another member of the hnRNPs family, synaptotagmin-binding cytoplasmic RNA-interaction protein (SYNCRIP), also participates in the sorting of miRNAs into exosomes by specifically binding to miR-3470a and miR-194-2-3p (Santangelo et al., 2016). Interestingly, hnRNPK, which is usually known to bind mRNA, was recently found to interact with miR-148a-3p via the AsUGnA motif (Robinson et al., 2021).

Fragile X mental retardation 1 (FMR1) was found to help control exosomal miRNAs’ loading during inflammation. Ann L. Wozniak and colleagues showed that overexpression of FMR1 resulted in the enrichment of miR-155 in EVs. Furthermore, the immunoprecipitation of FLAG-tagged rab-interacting lysosomal protein (RILP) constructs showed that cleaved RILP fragment (cRILP) interacted with Hrs, and when RILP was cleaved into cRILP by using LPS/ATP, the interaction of FMR1 and Hrs increased. These experiments suggested that cRILP recruit FMR1 to the MVBs to regulate the loading of miR-155 (Wozniak et al., 2020).

Besides, Y-Box binding protein 1 (YBX-1) was reported to be involved in sorting miR-223 into exosomes through liquid chromatography and mass spectrometry (Shurtleff et al., 2016; Shurtleff et al., 2017). Another study by Fengxia Lin et al. showed that YBX-1 could mediate the sorting of miR-133 into hypoxia/reoxygenation-induced endothelial progenitor cell (EPC)-derived exosomes as well (Lin et al., 2019). Moreover, the silencing of YBX-1 reduced the localization of exosomal miR-133, and vice versa (Lin et al., 2019).

Major vault protein (MVP) is a kind of ribonucleoprotein that mainly locates in the cytoplasm and associates with the cytoskeleton, and a small amount is around the nuclear membrane and pore complex (Wang W. et al., 2020). Recent research works imply the participation of MVP in the sorting of exosomal miRNAs. In colon cancer cells, MVP knockout resulted in decrease of exosomal miR-193a. Immunoprecipitation and qRT-PCR results further indicated that MVP is a potential binding partner of miR-193a (Teng et al., 2017).

#### 2.3.2 Membrane Proteins

Neural sphingomyelinase 2 (nSMase2) is a hydrolase enzyme that involved in sphingolipid metabolism reactions (Sindhu et al., 2021). Nobuyoshi Kosaka et al. demonstrated that inhibiting the activity of nSMase2 lead to reduced exosomal miRNA secretion. By contrast, overexpression of nSMase2 generated a higher exosomal miRNA level (Kosaka et al., 2010; Kosaka et al., 2013). Further experiments suggested that inhibiting nSMase2 with a selective inhibitor GW4869 resulted in a decrease of miR-210 in exosomes from mesenchymal stem cells (MSCs) (Zhu et al., 2018).

Caveolin-1 (Cav-1) is the major resident scaffolding protein that acts on forming caveolae. It was recently found that deletion of Cav-1 reduced the content of hnRNPA2B1 both in cells and microvesicles (MVs). Furthermore, the levels of hnRNPA2B1-binding miR-17, miR-93 were also decreased (Lee et al., 2019). Meanwhile, Cav-1 also regulated hnRNPK subcellular localization. The expression of Cav-1 resulted in the translocation of hnRPK to the MVB (Robinson et al., 2021). Since hnRNPA2B1 and hnRNPK both act an important role in the sorting of exosomal miRNA, it is obvious that Cav-1 performs a pivotal role in selective transport of miRNAs into EVs.

Vacuolar protein sorting 4 (Vps4) is important in the sorting of MVBs by triggering constriction and cleavage of ESCRT-III helical filaments (Maity et al., 2019). After inhibiting the expression of Vps4A in HEK293 cells, the amount of miR-150 and miR-92a was downregulated (Jackson et al., 2017). Meanwhile, overexpression of Vps4A in hepatocellular carcinoma (HCC) cells induced the selection of exosomal miR-193a-3p, miR-320a, miR-132-3p, miR-27b-3p and miR-92a-3p, probably through PI3K/Akt axis (Wei et al., 2015). These results suggested an important role of Vps4 in the selection of exosomal miRNAs. Since miR-27b-3p and miR-92a-3p are both significantly associated with tumor progression (Li et al., 2019; Yang et al., 2019), some potential treatment targets may be found among Vps4 in the future.

## 3 Role of Exosomal MicroRNA in Cancer Hallmarks

**FIGURE 2:**
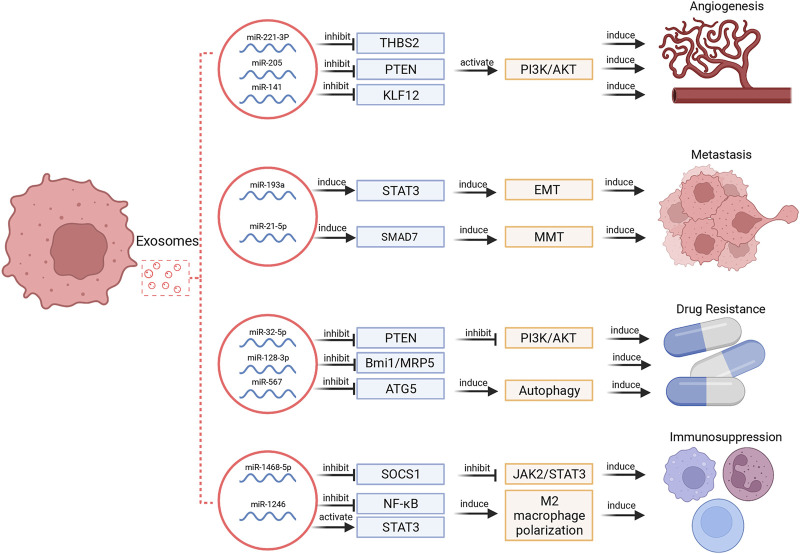
Exosomal miRNAs regulate different hallmarks of cancers. These functions include regulation of angiogenesis, metastasis, drug resistance and immunosuppression.

Tumor cells may secrete much more exosomes than normal cells, and tumor-derived exosomes can enroll in the cancer development by facilitate local and distant communication between cells via transporting multiple biomolecules (Akers et al., 2013; Sun et al., 2018). Among these biomolecules, miRNAs are extremely important in tumor development and invasion. The multifaceted functions of exosomal miRNAs in cancer hallmarks were illustrated in Figure 2.

### 3.1 Angiogenesis

Tumor growth depends on angiogenesis to a large extent. Exosomal miRNAs selected from several types of cells, such as MSCs, tumor cells, stromal cells, and endothelial cells can promote angiogenesis (Sun et al., 2018). For instance, exosomes secreted by MSCs can modulate endothelial tip cell specification via targeting on the 3′UTR of Delta-like ligand 4 (DLL4) to induce angiogenesis (Liang et al., 2016). Small cell lung cancer-derived exosomal miR-141 induced neovascularization through targeting Kruppel-like factor 12 (KLF12) (Mao et al., 2020). In ovarian cancer, tumor cell-derived miR-205 modulated angiogenesis by regulating the PTEN/Akt pathway (He et al., 2019). In cervical squamous cell carcinoma, cancer-derived exosomal miR-221-3p can directly bind to the 3′ UTR region of thrombospondin 2 (THBS2) mRNA to decrease its expression and thereby provoke angiogenic effect (Wu et al., 2019).

Certain conditions of cellular stress like hypoxia are reported to alter the miRNA composition of exosomes, modulate tumor microenvironment and promote angiogenesis (Panvongsa et al., 2022). In lung cancer, hypoxic tumor cells secret more exosomes than normoxic cells. Exosomal miR-23a was upregulated in hypoxic cancer cells, which could inhibit expression of prolyl hydroxylases (PHD1, 2) and tight junction protein zonula occludens (ZO-1) to increase angiogenesis in recipient vascular endothelial cells (Hsu et al., 2017). Similarly, hypoxic glioblastoma cell-derived exosomal miR-182-5p facilitated angiogenesis via silencing transcription factor KLF2 and KLF4 (Li J. et al., 2020).

On the contrary, some exosomal miRNAs can exhibit inhibitory effect on angiogenesis as well. Expression of miR-9 was obviously reduced in exosomes derived from nasopharyngeal carcinoma (NPC) cells and plasma samples. Moreover, exosomal miR-9 expression was negatively correlated with microvessel density in NPC, indicating a potential inhibitory role of exosomal miR-9 in angiogenesis. Further studies suggested that this process was mediated by directly targeting MDK and regulating phosphoinositide-dependent kinase-1 (PDK1)/Akt pathway (Lu J. et al., 2018).

### 3.2 Tumor Metastasis

The induction of metastasis *via* exosomal miRNAs has been well documented in various cancers. In gastric cancer, exosomal miR-21-5p promoted peritoneal metastasis via directly targeting SMAD7-3′-UTR and inducing mesothelial-to-mesenchymal transition (MMT) (Li Q. et al., 2018). In HCC, interference of exosomal miR-374a-5p inhibited cell metastasis (Lin et al., 2020). Epithelial mesenchymal transformation (EMT) is characterized as a conservative process in which epithelial-like tumor cells acquire mesenchymal properties and tend to be more aggressive and invasive (Shi et al., 2021). Accumulative evidences show that EMT is involved in initiation of tumor cells’ metastasis (Campbell, 2018). In lung cancer, transfer of exosomal miR-193a, miR-210-3p, miR-5100 from hypoxic bone marrow MSCs promoted cancer cells’ invasion *via* signal transducer and activator of transcription 3 (STAT3) driven EMT (Zhang et al., 2019).

Generally, exosomal miRNAs may induce tumor metastasis in several ways. First, less aggressive tumor cells can receive exosomes from donor cells with higher aggressivity, which may promote primary tumor progression. For example, highly metastatic breast tumor cell-derived exosomal miR-9 and miR-155 can induce metastasis in non-metastatic cells via targeting phosphatase and tensin homologue (PTEN) and dual specificity protein phosphatase 14 (DUSP14), respectively (Kia et al., 2019). Second, primary tumor cells can communicate with other cellular components *via* exosomal miRNAs to form pre-metastatic niche. For instance, breast tumor cell-derived exosomal miR-21 could regulate osteoclastogenesis to form pre-metastatic microenvironment for bone metastasis (Yuan et al., 2021). In colorectal cancer (CRC), exosomal miR-934 induced M2 polarization of macrophages via downregulating PTEN and activating PI3K/Akt to promote liver metastasis (Zhao et al., 2020). Fibroblasts are spindle-shaped cells that synthesize collagen in connective tissue, and cancer-associated fibroblasts are activated fibroblasts that are found to play a role in aggravated tumor progression (Li YY. et al., 2018). Exosomal miRNAs in cancer-associated fibroblasts can also induce or restrain tumor cells’ metastasis. For example, cancer-associated fibroblast-derived exosomal miR-34a-5p and miR-382-5p contributed to oral cancer cells’ proliferation/metastasis or cell motility/invasiveness, respectively (Li YY. et al., 2018; Sun et al., 2019), while exosomal miR-139 in cancer-associated fibroblasts inhibited gastric cancer progression via repressing matrix metalloproteinase 11 (MMP11) expression (Xu et al., 2019).

### 3.3 Drug Resistance

Drug resistance, especially chemoresistance, is one of the most challenging issues in the treatment of cancer patients (Tu et al., 2020a; Tu et al., 2020b; Lohan-Codeco et al., 2022). It can be induced by several biological determinants including tumor burden, growth kinetics, tumor heterogeneity, genomic drivers, and tumor microenvironment (Vasan et al., 2019). Since exosomal miRNAs participate in tumor metastasis and tumor niche formation, a growing body of investigations have focused on the role of exosomal miRNAs in neoplastic drug resistance. Among them, tumor-derived exosomes, macrophage-derived exosomes (MDEs) and cancer-associated fibroblast-derived exosomal miRNAs are strongly correlated with drug resistance.

Tumor-derived exosomes are reported to participate in both reversal and induction of drug resistance. In breast cancer, overexpression of miR-567 could promote trastuzumab-induced cell death, while silencing miR-567 decreased cell death, suggesting that miR-567 promotes chemosensitivity of trastuzumab treatment. Mechanistically, miR-567 was incorporated into tumor-derived exosomes and reversed drug resistance via inhibiting autophagy-related 5 (ATG5) expression (Han et al., 2020). Besides, exosomal miR-128-3p could increase chemosensitivity to oxaliplatin in CRC by negatively regulating expression of Bmi1 and multidrug resistance (MDR) protein 5 (MRP5) (Liu et al., 2019b). On the other hand, tumor-derived exosomes can induce cancer cell drug resistance. In non- small cell lung cancer (NSCLC), gemcitabine resistant cells transferred drug resistance via selecting exosomal miR-222-3p (Wei et al., 2017). In HCC, miR-32-5p was delivered from resistant cells to sensitive cells through exosomes, activated the PI3K/Akt pathway by downregulating PTEN, and thus modulated angiogenesis and EMT to induce MDR (Fu et al., 2018).

Exosomal miRNAs derived by tumor-associated macrophages (TAMs) mostly accompany in inducing drug resistance. Cytidine deaminase (CDA) is the major enzyme of gemcitabine inactivation since overexpressed CDA converses into dFdUridine to inactivate gemcitabine. In pancreatic adenocarcinoma (PDAC), transfer of miR-365 in MDEs induced gemcitabine resistance via modulating pyrimidine metabolism and CDA expression (Binenbaum et al., 2018). In gastric cancer, TAM-derived exosomal miR-21 induced cisplatin resistance. Importantly, PTEN/PI3K/Akt pathway was found to be associated with the cisplatin resistance induction (Zheng et al., 2017), suggesting a potential therapeutic target in this pathway.

Cancer-associated fibroblasts can co-evolve with tumor cells and help to form tumor niche, and thereby contribute to tumor progression (Chen and Song, 2019). Cancer-associated fibroblast-derived exosomal miRNAs play an important role in induction of drug resistance. In gastric cancer, a novel mechanism of acquired chemoresistance through USP7/hnRNPA1/miR-522/ALOX15 axis was identified. Firstly, chemotoxicity increased USP7 expression and reduced ubiquitination of hnRNPA1. Overexpressed USP7 then stabilized hnRNPA1 in cancer-associated fibroblasts via deubiquitination and enhanced secretion of exosomal miR-522. Finally, exosomal miR-522 directly targeted ALOX15 in gastric cells to restrain lipid- reactive oxygen species (ROS) production and ferroptosis, resulting in acquired chemoresistance (Zhang et al., 2020a). In breast cancer, CD63^+^ cancer-associated fibroblast-derived miR-22 promoted tamoxifen resistance via targeting PTEN and estrogen receptor 1 (ESR1) (Gao et al., 2020).

### 3.4 Immunosuppression

Exosomal miRNAs are critical mediators in the communication between tumor cells and immune cells. Tumor-derived exosomal miRNAs have been proven to affect the structure of macrophages and arise immunosuppression. For example, TP53 mutants are correlated with pathogenesis of a variety of human cancers, and mutp53 can reprogram macrophages into tumor supporting macrophages *via* exosomal miR-1246 to induce an anti-inflammatory microenvironment, recruit immunosuppressive Treg cells and promote tumor progression (Cooks et al., 2018). In addition to macrophages, tumor-derived exosomal miRNAs can also induce dysfunction of immune cells to facilitate immunosuppression. Exosomal miR-1468-5p derived by tumor-derived exosomes directly targeted homeobox containing 1 (HMBOX1) in human dermal lymphatic endothelial cell (HDLEC) and inhibited suppressor of cytokine signaling 1 (SOCS1) expression to activate the JAK2/STAT3 pathway and mediate HDLEC reprogramming to repress CD8^+^ T cell immunity (Zhou et al., 2021).

## 4 Exosomal MicroRNA in Osteosarcoma

Osteosarcoma is one of the most common pediatric malignancies with low morbidity but high lethality. Much emphasis is rightly placed on management of osteosarcoma, but efficient management is still on the way due to the existence of unusual inter- and intratumoral heterogeneity within osteosarcoma (Whelan and Davis, 2018). Since exosomal miRNAs are found to exert critical effects in tumor development, the functions of miRNAs in osteosarcoma have attracted a great attention. A set of miRNAs have been found to show excellent diagnostic potential in osteosarcoma, such as miR-195-5P, miR-302a, miR-374a-5p and miR-152 (Gally et al., 2021). More recently, a series of evidences demonstrated that exosomal miRNAs could participate in osteosarcoma progression (Table 1).

**TABLE 1:** Exosomal miRNAs involved in osteosarcoma progression.

Exosomal miRNAs	Targets	Functions	References
miR-148a-3p	/	Promote angiogenesis	Raimondi et al., (2020)
miR-21-5p	/	Promote angiogenesis	Raimondi et al., (2020)
miR-25-3p	DKK3	Promote angiogenesis	Fujiwara et al., (2017)
miR-1228	SCAI	Promote metastasis	Wang et al., (2019)
miR-221-3p	SOCS3	Promote metastasis	Liu et al., (2021)
miR-675	CALN1	Promote metastasis	Gong et al., (2018)
miR-1307	AGAP1	Promote metastasis	Han et al., (2021)
miR-208a	PDCD4	Promote metastasis	Qin et al., (2020)
miR-206	TRA2B	Inhibit metastasis	Zhang et al., (2020b)
miR-15a	GATA2	Inhibit cell cycle	Wu et al., (2021)

AGAP1, ankyrin repeat and PH domain 1; CALN1, calneuron 1; DKK3, dickkopf-3; GATA2, GATA binding protein 2; PDCD4, programmed cell death protein 4; SCAI, suppressor of cancer cell invasion; SOCS3, suppressor of cytokine signaling 3; TRA2B, transformer 2 beta homolog.

### 4.1 Exosomal miRNAs That Promote Osteosarcoma Progression

Recently, exosomal miRNAs have been shown to substantially contribute to tumor progression in multiple malignancies. Among them, there are eight exosomal miRNAs that proved to promote the progression of osteosarcoma.

#### 4.1.1 Exosomal miR-148a-3p and miR-21-5p

Exosomal miR-148a-3p and miR-21-5p have been found to participate in the pathogenesis of several cancers. In glioma, tumor-derived exosomal miR-148a-3p promoted endothelial cell proliferation and angiogenesis via inhibiting ERRFl1 and activating the EGFR/MAPK pathway (Wang M. et al., 2020). In CRC, tumor-secreted exosomal miR-21-5p downregulated KR1T1 expression and mediated the β-catenin pathway to induce vascular permeability and angiogenesis (He Q. et al., 2021).

As to osteosarcoma, both miR-148a-3p and miR-21-5p were found to be downregulated in tumor-derived exosomes and can interfere with osteoclast and endothelial cells’ activity, stimulate the release of proangiogenic factors, and induce vessel formation, indicating a significant correlation with tumor progression (Raimondi et al., 2020).

#### 4.1.2 Exosomal miR-25-3p

MiR-25-3p was found to be upregulated in exosomes of several cancers like liposarcoma and CRC. In liposarcoma, miR-25-3p was secreted by tumor cells into blood vessels and involved in the communication between tumor cells and microenvironment (Casadei et al., 2017). In CRC, tumor cell-secreted exosomal miR-25-3p can directly silence KLF2 and KLF4 to downregulate the expression of vascular endothelial growth factor receptor 2 (VEGFR2), ZO-1, occludin and Claudin5 in endothelial cells and finally induce angiogenesis and vascular permeability (Zeng et al., 2018).

In osteosarcoma, miR-25-3p is upregulated in exosomes of multiple cell lines, and clinicopathological analysis showed that the serum mR-25-3p expression was positively correlated with osteosarcoma progression, suggesting that serum exosomal miR-25-3p may serve as a new diagnostic and prognostic biomarker in osteosarcoma (Fujiwara et al., 2017).

#### 4.1.3 Exosomal miR-1228

MiR-1228 was found to be dysregulated in exosomes in several kinds of cancers. In NCSLC, exosomal miR-1228 expression was decreased as compared with healthy controls (Xia et al., 2021). In gastric cancer, miR-1228 was downregulated in exosomes as well. From a molecular perspective, exosomal miR-1228 secreted by human bone marrow MSCs regulated the growth of gastric cancer via targeting MMP-14 (Chang et al., 2021).

By contrast, miR-1228 was dramatically increased in the exosomes derived by cancer-associated fibroblasts in osteosarcoma. These exosomes with increased miR-1228 were internalized by tumor cells and then promoted the osteosarcoma migration and invasion. Mechanistic assays showed that exosomal miR-1228 led to this promotion by directly targeting suppressor of cancer cell invasion (SCAI) (Wang et al., 2019).

#### 4.1.4 Exosomal miR-221-3p

Exosomes miR-221-3p has been identified to participate in the metastasis and drug resistance in various cancers. For instance, in cervical squamous cell carcinoma, miR-221-3p in exosomes released by tumor cells can downregulate vasohibin-1 (VASH1) and activate Akt and ERK pathway to induce lymphangiogenesis and lymphatic metastasis (Zhou CF. et al., 2019). In breast cancer, exosomal miR-221-3p secreted by cancer cells can downregulate phosphoinositide-3-kinase regulatory subunit 1 (PIK3R1) and inhibit the PI3K/Akt signaling pathway to promote adriamycin resistance (Pan et al., 2020).

As to osteosarcoma, pulmonary metastasis is the leading cause of death (He J. et al., 2021; Zhang C. et al., 2021; Zhang W. et al., 2021). MiR-221-3p was found to be overexpressed in exosomes secreted by M2-polarized TAMs and these exosomes intensified the malignant progression of osteosarcoma. Further studies revealed that exosomal miR-221-3p can inhibit suppressor of cytokine signaling 3 (SOCS3) and activate the JAK2/STAT3 signaling pathway to promote the growth and metastasis of osteosarcoma (Liu et al., 2021).

#### 4.1.5 Exosomal miR-675

MiR-675 was upregulated in exosomes secreted by metastatic osteosarcoma cells. These exosomes mediated the transfer of miR-675 from osteosarcoma cells to stromal cells, and miR-675 downregulated the expression of calneuron 1 (CALN1), resulting in the promotion of migration and invasion of tumor cells (Gong et al., 2018).

#### 4.1.6 Exosomal miR-1307

Exosomal miR-1307 was highly expressed in serum of ovarian cancer patient compared to benign or healthy counterparts. Its expression was obviously correlated with tumor staging. More importantly, it could be used as independent diagnostic tool with enhanced accuracy when compared with conventional biomarkers, such as CA-125 and HE4 (Su et al., 2019).

More recently, miR-1307 was found to be abundantly expressed in osteosarcoma exosomes and cells, as well as human osteosarcoma tissues. Among them, tumor cell-derived exosomal miR-1307 induced the cell proliferation, migration and invasion. Mechanistic assays showed that miR-1307 can directly bind to the 3′-UTR of ArfGAP with GTPase domain, ankyrin repeat and PH domain 1 (AGAP1) and inhibit its expression to promote tumorigenesis (Han et al., 2021).

#### 4.1.7 Exosomal miR-208a

MiR-208a has a close relationship with tumor metastasis and radio-resistance. In lung cancer, miR-208a was significantly upregulated in serum after radiotherapy, and serum miR-208a was translocated into lung cancer cells via exosomes. Further studies showed that miR-208a may directly bind to the 3′UTR of p21 to reduce its expression and activate the Akt/mTOR pathway. Consequently, exosomal miR-208a enhanced the proliferation and radio-resistance of lung cancer (Tang et al., 2016).

While in osteosarcoma, exosomal miR-208a secreted by bone marrow MSCs was found to promote the viability, clonogenicity and migration of osteosarcoma cells. Furthermore, exosomal miR-208a inhibited programmed cell death protein 4 (PDCD4) and then activated ERK1/2 signaling pathway to enhance the aggressiveness of osteosarcoma (Qin et al., 2020).

### 4.2 Exosomal miRNAs That Inhibit the Progression of Osteosarcoma

Till now, only two exosomal miRNAs were found to inhibit the progression of osteosarcoma, and they may show the potential in the treatment of osteosarcoma in the future.

#### 4.2.1 Exosomal miR-206

MiR-206 has been found to be closely related to the occurrence and progression of several kinds of cancers. In NSCLC, miR-206 can inhibit the expression of coronin 1C (CORO1C) to negatively regulate the tumor metastasis (Liao and Peng, 2020). In intrahepatic cholangiocarcinoma, exosomal miR-206 reduced the expression of LIM and SH3 protein 1 (LASP1) and suppressed the activity of STAT3 signaling to inhibit cholangiocarcinoma stem-like characteristics and TGF-β1 secretion. Additionally, miR-206 can further inhibit the interactions between tumor cells and stromal cancer-associated fibroblasts to suppress tumor deterioration and promote sensitivity to gemcitabine (Yang et al., 2022).

**FIGURE 3:**
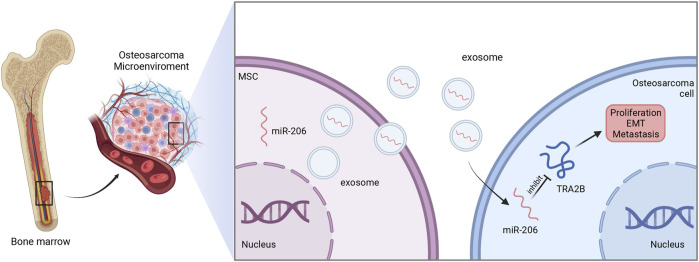
The function of exosomal miR-206 in osteosarcoma. Bone marrow MSCs-derived miR-206 is wrapped and transported to osteosarcoma cells by exosomes, and in turn suppresses TRA2B to inhibit the proliferation, EMT and metastasis of osteosarcoma.

In osteosarcoma cell, miR-206 was poorly expressed. Exosomal miR-206 derived by bone marrow MSCs can be internalized by osteosarcoma cells. Dual-luciferase reporter gene assay further showed that miR-206 can directly bind to 3′-UTR of transformer two beta homolog (TRA2B), and thereby suppress its expression. Since TRA2B is associated with EMT-related proteins (twist, N-cadherin, and E-cadherin), miR-206 encapsulated in exosomes could also impede the osteosarcoma metastasis by downregulating TRA2B-induced EMT. Moreover, functional assays revealed that exosomal miR-206 could facilitate osteosarcoma cell apoptosis, and inhibit proliferation, migration, and invasion (Figure 3). Accordingly, alteration of miR-206 expression might be a potential therapeutic strategy in osteosarcoma treatment (Zhang et al., 2020b).

#### 4.2.2 Exosomal miR-15a

MiR-15a is located at 13q14.3 of the human genome, and acts as a tumor suppressor in different types of cancers (Huang et al., 2015; Liu et al., 2019a; Wu et al., 2021). In HCC, MSCs-derived exosomes transferred miR-15a to target cells and then suppressed proliferation, migration, and invasion of tumor cells via inhibiting the expression of spalt like transcription factor 4 (SALL4) (Ma et al., 2021).

**FIGURE 4:**
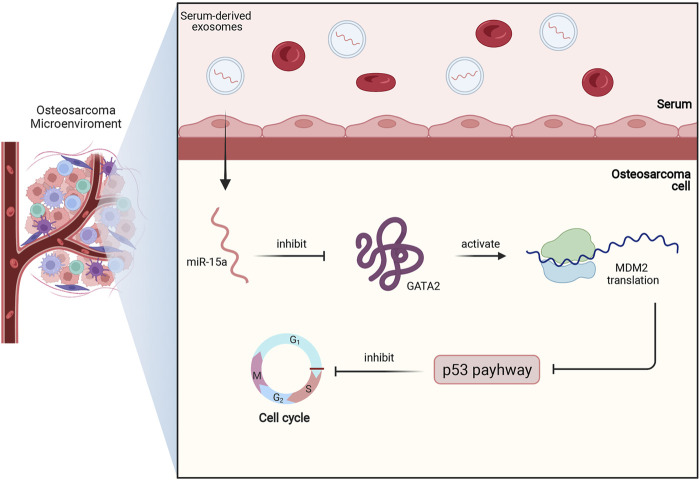
This figure briefly shows miR-15a/GATA2/MDM2/p53 axis in osteosarcoma. Firstly, miR-15a is transported to osteosarcoma cells by serum-derived exosomes, and then miR-15a directly binds to GATA2 and inhibits its expression. Subsequently, the downregulated expression of GATA2 downregulates the MDM2 translation and activates the p53 signaling pathway. Finally, the activated p53 pathway contributes to cell cycle arrest and apoptosis of osteosarcoma.

In osteosarcoma cell, miR-15a was also found to be downregulated. Moreover, low expression of miR-15a indicated an unfavorable survival rate in osteosarcoma patients. Further assays showed that serum-derived exosomes with miR-15a can be internalized by osteosarcoma cells and then miR-15a directly binds to GATA binding protein 2 (GATA2) to inhibit its expression. Since GATA2 can induce the transcription of murine double minute 2 (MDM2) to suppress the p53 signaling pathway, serum-derived exosomal miR-15a can promote apoptosis and cell cycle arrests of osteosarcoma cells via GATA2/MDM2/p53 axis, as demonstrated in Figure 4 (Wu et al., 2021).

### 4.3 Interaction With Signaling Pathways

It is well known that PI3K/Akt, p53, Wnt, Notch, NF-κB and MAPK are important pathways in osteosarcoma pathogenesis (Czarnecka et al., 2020). A plethora of studies have expanded our understanding of the interplay between exosomal miRNAs and some signaling pathways in osteosarcoma. For instance, miR-122-5p was found to modulate the activation of PI3K/Akt/mTOR signaling pathway via targeting TP53 to regulate cell proliferation and apoptosis (Li KW. et al., 2020). In osteosarcoma, exosomal miR-15a was reported to downregulate GATA2 and inhibit MDM2, which activates p53 pathway to induce cell cycle arrest (Wu et al., 2021). MiR-25-3p directly targeted dickkopf-3 (DKK3), which acted on an important part in the Wnt-β catenin pathway (Yoshida et al., 2018).

However, the crosstalk of exosomal miRNAs with Notch, NF-kB and MAPK pathways have not been identified in osteosarcoma yet. Of note, only studies concerning the interaction between miRNAs and these pathways were reported. MiR-338-3p targeted RNX2/CDK4 and inhibited MAPK pathway to suppress tumor growth (Jia et al., 2019). MiR-1296-5p targeted Notch2 to suppress the proliferation, migration, and invasion of osteosarcoma (Wang L. et al., 2020). MiR-155 regulated osteosarcoma progression via regulating NF-kB pathway (Lu S. et al., 2018). Similarly, hsa-let-7g reduced HOXB1 to activate NF-kB pathway to promote osteosarcoma progression (Zhou JL. et al., 2019). More studies with regard to the pivotal role of exosomal miRNAs in regulation of these pathways may be explored in near future.

### 4.4 Clinical Applications of Exosomal miRNAs in Osteosarcoma

Circulating exosome miRNAs may be promising cancer biomarkers. First, it requires only noninvasive or minimally invasive procedure and could be easily accessed through body fluid, most frequently peripheral blood. Second, it is repeatable and inexpensive. Third, it could offer real-time information and thus reflect the tumor cell evolution (Panvongsa et al., 2022). Fourth, miRNAs inside the exosome are more stable since the phospholipid bilayer membrane of exosomes can protect miRNAs from the degradation by RNase (Wang et al., 2022). Thus, exosomal miRNA-based biomarkers in liquid biopsies may open up novel possibilities for cancer screening, early diagnosis, and monitoring (Panvongsa et al., 2022).

Recently, some studies have explored the potential diagnostic and therapeutic values of dysregulated exosomal miRNAs in osteosarcoma (Lima et al., 2009). Yoshida et al. showed that extracellular miR-25-3p was enriched in exosome, and its expression was significantly associated with poor prognosis of osteosarcoma patients (Yoshida et al., 2018). Another study performed by Lavinia Raimondi et al. explored the different expression pattern of miRNAs between osteosarcoma cell lines and derived exosomes by small RNA sequencing. Data analysis showed that there are 21 miRNAs with remarkable difference. Among the 21 miRNAs, ten were downregulated in exosomes (hsa-let-7b-5p, hsa-let-7d-3p, hsa-let-7e-5p, hsa-miR-23a-5p, hsa-miR-214-3p, hsa-miR-125a-5p, hsa-miR-331-3p, hsa-miR-193b-3p, hsa-miR-941 and hsa-miR-1908-5p), while eleven were upregulated in exosomes (hsa-let-7f-5p, hsa-miR-16-5p, hsa-miR-21-5p, hsa-miR-192-5p, hsa-miR-148a-3p, hsa-miR-182-5p, hsa-miR-128-3p, hsa-miR-126-5p, hsa-miR-186-5p, hsa-miR-301a-3p and hsa-miR-151a-3p) (Raimondi et al., 2020). By dissecting the exosomal miRNA profile, the potential role for these miRNAs as prognostic biomarkers in osteosarcoma may also been revealed.

## 5 Conclusion and Future Perspective

Exosomes could be released by a variety of types of cells and convey bioactive cargoes to modulate crosstalk between donor and recipient cells. MiRNAs are selectively sorted into exosomes as one of the essential components (Wang et al., 2022). As aforementioned, various carcinogenesis-associated exosomal miRNAs are aberrantly expressed in osteosarcoma, and are proven to be markedly correlated with its onset and progression. Exosomal miRNAs could regulate osteosarcoma cell growth, migration, angiogenesis and metastasis by guiding degradation of target mRNA or interplaying with other signaling pathways including p53, PI3K/AKT/mTOR and Wnt-β catenin (Shi et al., 2021). This indicates that reversing abnormally expressed exosomal miRNA may be a novel therapeutic approach in osteosarcoma. However, several questions remained to be elucidated yet.

First, some aspects regarding the mechanism of exosomal miRNAs in drug resistance and immune disturbance in osteosarcoma remain unclear. Up to now, only studies on the association between miRNAs or other bioactive substances in exosome and drug resistance are noted in osteosarcoma. For example, miR-15b can modulate MDR in human osteosarcoma both *in vitro* and *in vivo*. MiR-15b reconstitution can reverse chemotherapy resistance in osteosarcoma (Duan et al., 2017). Similarly, miR-221 was found to induce cisplatin resistance in osteosarcoma via targeting PI3K/Akt pathway (Zhao et al., 2013). Besides, MG-63DXR30 cells may transmit doxorubicin resistance to osteosarcoma MG-63 cells by released exosomes. However, the specific active cargoes were MDR-1 mRNA and its product P-glycoprotein rather than miRNAs (Torreggiani et al., 2016). Currently, the relationship between exosomal miRNAs and drug resistance or immunosuppression has not been demonstrated, which are worthy of in-depth study.

Second, it should be noted that most of previous studies on exosomal miRNAs in osteosarcoma are based on *in vitro* assay. While animal studies or multi-center, large clinical cohort are still lacking. Therefore, more *in vivo* experiments or clinical studies with large sample size are still necessary for further validation.

Last, the aberrant expression of exosomal miRNAs may be detected for efficient clinical diagnosis or prognosis prediction. Recently, tremendous abnormally expressed exosomal miRNAs have been identified in osteosarcoma due to the advancement of high-throughput NGS. However, only a few of these miRNAs have been annotated and extensively studied. While other miRNAs remain unexplored. Despite a set of breakthroughs have been made in this field, more rigid studies are still needed to further unveil the specific clinicopathologic significance and underlying mechanism of exosomal miRNAs in osteosarcoma.
